# Older Aboriginal Australians’ Health Concerns and Preferences for Healthy Ageing Programs

**DOI:** 10.3390/ijerph17207390

**Published:** 2020-10-10

**Authors:** Pamela Ming Wettasinghe, Wendy Allan, Gail Garvey, Alison Timbery, Sue Hoskins, Madeleine Veinovic, Gail Daylight, Holly A. Mack, Cecilia Minogue, Terrence Donovan, Gerald A. Broe, Kylie Radford, Kim Delbaere

**Affiliations:** 1 UNSW Medicine, University of New South Wales, Kensington, NSW 2052, Australia; pam.wettasinghe@gmail.com (P.M.W.); t.broe@neura.edu.au (G.A.B.); k.delbaere@neura.edu.au (K.D.); 2 Aboriginal Health and Ageing Program, Neuroscience Research Australia, Randwick, NSW 2031, Australia; w.allan@neura.edu.au (W.A.); a.timbery@neura.edu.au (A.T.); sdhoskinssuehoskinssdhoskins@outlook.com (S.H.); m.veinovic@neura.edu.au (M.V.); cliffdaylight@gmail.com (G.D.); cecilia.minogue@icloud.com (C.M.); t.donovan@neura.edu.au (T.D.); 3 Wellbeing and Preventable Chronic Diseases Division, Menzies School of Health Research, Brisbane, QLD 4000, Australia; gail.garvey@menzies.edu.au; 4 Faculty of Health, University of Technology, Sydney, NSW 2007, Australia; Holly.Mack@uts.edu.au; 5 UNSW Ageing Futures Institute, University of New South Wales, Kensington, NSW 2052, Australia

**Keywords:** indigenous population, ageing, health promotion, healthcare access, dementia, chronic disease, falls, technology, physical activity

## Abstract

While there is strong evidence of the need for healthy ageing programs for older Aboriginal Australians, few are available. It is important to understand older Aboriginal Australians’ perspectives on healthy ageing in order to co-design culturally-appropriate programs, including views on technology use in this context. Semi-structured interviews were conducted with 34 Aboriginal Australians aged 50 years and older from regional and urban communities to explore participants’ health concerns, preferences for healthy ageing programs, and receptiveness to technology. Qualitative data were analyzed using a grounded theory approach. This study found that older Aboriginal Australians are concerned about chronic health conditions, social and emotional well-being, and difficulties accessing health services. A range of barriers and enablers to participation in current health programs were identified. From the perspective of older Aboriginal people, a successful healthy ageing program model includes physical and cognitive activities, social interaction, and health education. The program model also provides culturally safe care and transport for access as well as family, community, cultural identity, and empowerment regarding ageing well as central tenets. Technology could also be a viable approach for program delivery. These findings can be applied in the implementation and evaluation of culturally-appropriate, healthy ageing programs with older Aboriginal people.

## 1. Introduction

Australia’s Indigenous peoples comprise two distinct cultural groups: Aboriginal and Torres Strait Islander peoples. Aboriginal peoples are of diverse Aboriginal nations, who have historically lived on mainland Australia, Tasmania, or the continent’s offshore islands. Torres Strait Islander people come from the islands of the Torres Strait and are of Melanesian origin. Aboriginal and Torres Strait Islander peoples (hereafter referred to as Aboriginal Australians) currently comprise 3% of the Australian population [1]. While improvements have been achieved in many aspects of Aboriginal Australians’ health and well-being, significant disparities between Aboriginal and non-Indigenous Australians remain. Life expectancy is approximately 10 years lower [2], and multi-morbidity is 2.59 times more prevalent in Aboriginal than non-Indigenous Australians [3]. For many Aboriginal people, healthy ageing may be more difficult to attain in this context. Yet, the life expectancy of Aboriginal Australians continues to increase [2], and the number of Aboriginal Australians over age 65 is expected to almost triple from 22,700 people in 2011 to 61,900 people in 2026 [4]. There is, hence, an increasing need for appropriate programs for older Aboriginal Australians to support their health, well-being, and quality of life.

As expressed in the National Aboriginal Health Strategy (NAHS) [5], health is defined holistically, beyond just physical health or functional capacity, to include ‘the social, emotional, and cultural well-being of the whole community in which each individual is able to achieve their full potential as a human being, thereby, bringing about the total well-being of their community. It is a whole-of-life view and includes the cyclical concept of life-death-life.’ Consistent with this view is the recognition of the impact behind the social determinants of health on the well-being of Aboriginal people across the life course, including older age [6]. Inequalities in health arise from many interconnected social factors, which are determined by the circumstances in which people grow, live, work, and age [7]. Aboriginal Australians are not only impacted by generalized social determinants such as education, employment, income, and transport [8,9,10], but are also negatively impacted by cultural determinants, such as colonization, racism, loss of language, and loss of connection to land [11,12].

### 1.1. The Significance of Community Collaboration

Since Aboriginal Australians often experience a range of chronic health conditions throughout their lifespan [13], and in the context of the health, social, and cultural disparities they face, their needs as older people are likely to be more complex. Existing healthcare services tend not to consider the complex care or cultural safety needs of older Aboriginal Australians, resulting in overall poorer access to services [14,15,16]. To date, it is not well understood how these experiences have affected the perceived health care needs of this population [17].

Many older Aboriginal people are regarded as cultural custodians, mentors, and caregivers in their communities [17,18]. It is important to recognize the pivotal role they play in the health and well-being of their communities when designing a culturally-responsive, healthy ageing program. Coombes and colleagues [19] suggested that a culturally-appropriate, healthy ageing program addressing older Aboriginal Australians’ health needs and perceptions is imperative. The challenge now is developing and implementing such programs. Ottmann [20] describes a co-design approach, which places beneficiaries in positions of power and influence in the design process. This approach empowers Aboriginal Australians to shape health programs to suitably support their own needs [16,20].

Two examples of successfully co-designed health programs for older Aboriginal Australians are the Heart Health program [21] and the Ironbark program [22]. Established in 2009, Heart Health (Moorditj Koort) is an ongoing cardiac rehabilitation program at a metropolitan Aboriginal Medical Service (AMS) in Western Australia. It involves weekly exercise and education sessions for Aboriginal Australians with or at risk of cardiovascular disease. During its ‘consultation phase’, focus groups were conducted with Aboriginal Health Workers and community members to address relevant social determinants. This resulted in unique features, which set Heart Health apart from traditional hospital cardiac rehabilitation programs, such as the absence of a formal referral process, flexibility of attendance, having culturally safe meeting spaces, and an opportunity for ‘yarning’ (Aboriginal conversational method) during education sessions [21]. More recently, the Ironbark program was established as the first fall prevention program developed specifically for Aboriginal people [22]. It comprised a weekly program over three to six months including exercise and education components in which each was adapted from existing fall prevention programs developed for the general population.

Considering the limited evaluation of these programs across a range of measures, Heart Health and Ironbark have set the stage for further collaborative development of healthy ageing programs among older Aboriginal Australians. Furthermore, shifting the delivery of such programs onto technological platforms could be a valuable future direction, as discussed in the following section.

### 1.2. Technology as a Platform for Program Delivery

Older people face many barriers to participation in supervised center-based exercise programs, including lack of transport or locally available services, financial issues, or social embarrassment [23]. In addition to these social determinants, older Aboriginal people may face further cultural barriers. The delivery of programs via the Internet or on devices like tablets and smartphones via applications (apps.) has the benefits of overcoming barriers of distance and cost, improving flexibility of time and allowing relative anonymity [24,25].

Although Aboriginal Australians have been previously reported to have lower access to technology than non-Aboriginal Australians [26], recent research suggests that digital technologies are becoming increasingly commonplace in the lives of Aboriginal people. A national survey found that more than 60% of Aboriginal people are active social media users in remote areas [27]. The Information Technologies and Indigenous Communities (ITIC) symposiums in 2010 and 2017 also demonstrated a significant and diverse information technology (IT) sector within Aboriginal communities and revealed a variety of information technology used to support innovation, employment, training, and transmission of culture [28].

To date, there has been limited research on the co-design and evaluation of culturally-appropriate technology-based health approaches for older Aboriginal people. One study [29] explored Aboriginal experiences of using two e-mental health apps, which were developed through extensive community consultation–the AIMhi Stay Strong app (Menzies School of Health Research, Darwin, Australia) and the ibobbly suicide prevention app (Black Dog Institute, Sydney, Australia). Participants in these studies expressed strong support for the concept of an app. They also identified community collaboration to be a good approach for enhancing uptake, acceptability, and adherence of e-mental health apps. This aligns with the findings of others and reinforces the evidence for the co-design of e-Health tools [30,31].

### 1.3. Study Aims

Despite the strong evidence of the need for culturally-appropriate, healthy ageing programs and the potential viability of technology as a platform for program delivery, the translation of this evidence into practice among older Aboriginal Australians remains limited. The current study aimed to explore older Aboriginal Australians: (1) perceptions of healthy ageing and main health concerns, (2) preferred approaches to developing culturally-appropriate, healthy ageing programs, and (3) receptiveness to technology.

## 2. Materials and Methods

A participatory action research model was adopted for this project to develop and evaluate a novel program to support active and healthy ageing with older Aboriginal people. The current study represents the initial consultation and development stage. This approach “blurs the lines between the researcher and the researched through processes that accent the wealth of assets that community members bring to the process of knowing and creating knowledge and acting on that knowledge to bring about change” [32]. It requires an active engagement of those whose lives are impacted by the issue being researched in each stage of the study [33]. The study methods follow both the Consolidated Criteria for Reporting Qualitative Research (COREQ) [34] and Aboriginal and Torres Strait Islander Quality Appraisal Tool [35] reporting criteria.

### 2.1. Participant Recruitment

A convenience sample of Aboriginal and/or Torres Strait Islander men and women aged 50 years and older, living in regional (mid-north coast) and urban (greater Sydney) New South Wales (NSW), were recruited for this study. NSW is the most populated state of Australia, where ~30% of Aboriginal and Torres Strait Islander peoples reside [4], and participants were from areas where multiple health, aged care, and community services exist, including Aboriginal community-controlled health services. All participants were living independently in the community and were assumed to have capacity to consent to participate, unless concerns were identified to the contrary by the experienced researchers involved in this study (who also have clinical qualifications in nursing or psychology). Only those able to provide informed consent were included. Participants initially indicated an expression of interest after hearing about the project at community information sessions, via brochures distributed in local health organizations, or at information displays at local health promotion forums. A local community-based research assistant followed up expressions of interest with phone calls or emails to confirm their interest and participation in the study and, if they agreed, arranged a time for an interview. Participants were informed that this study followed on from the research team’s previous research [36] with older Aboriginal people in these communities, and that the researchers aimed to translate the current study’s findings into a culturally-appropriate, healthy ageing program that is relevant and beneficial to older Aboriginal people.

### 2.2. Data Collection

A single semi-structured interview was conducted with each participant from the regional communities in 2014 and from urban communities in 2017. The delay between regional and urban studies related to funding constraints. All consenting participants completed the interview. Questions for the semi-structured interviews were developed collaboratively within the research team, including Aboriginal community-based researchers (see Supplementary Material Table S1 for details of semi-structured interview questions). The Health Belief Model, a theory for understanding how beliefs and opinions can affect health behaviors [37], was applied to explore participants’:

- perceived susceptibility and severity of various health conditions,
- perceived benefits of and barriers to participation in healthy ageing programs,
- proposed strategies to activate readiness to adopt such programs,
- receptiveness to technology.

Interviews took place privately, at a local meeting room or other convenient places nominated by the local Aboriginal researchers or participants. Interviews were conducted by authors S.H. and A.T. (female research assistants from the local Aboriginal community), in conjunction with W.A. and C.M. (female postdoctoral researchers with extensive prior experience working with the participating communities, with clinical backgrounds in nursing and psychology, respectively). The duration of the interview ranged from 20 to 120 min.

### 2.3. Data Analysis

Interviews were audio-recorded and transcribed verbatim. Transcripts were coded using a grounded theory approach [38,39]. The transcripts were analyzed and closely examined for similarities and differences while looking for interrelated issues using the constant comparative method, which is a method used to develop grounded theory [38,39]. Though the study was not designed to generate theory, the structured approach of grounded theory was considered an appropriate methodology for researchers conducting research with a cultural group different from their own [38].

Throughout this process, authenticity and trustworthiness were key considerations. Criteria embedded in the research process to ensure trustworthiness were credibility, transferability, dependability, and conformability [40]. Strategies used to address these criteria are those described by Denzin and Lincoln [41] and previously used in qualitative studies [42].

The constant comparative method involved analyzing data across transcripts as well as comparing and grouping similar instances under the same conceptual label [39]. P.W. developed an excel spreadsheet for the compilation of recurring themes and comparison of concepts, accompanied by relevant quotes, across transcripts. Primary coding was completed by P.W. and W.A., and their data interpretations were reviewed and verified by both Aboriginal and non-Indigenous researchers within the project team, as well as through interactions with the participants where possible. Main themes and sub-themes were derived from the data (see Supplementary Material Table S2). Saturation of the main themes was achieved. An audit trail was established to trace quotes back to transcripts. For example, ‘Participant 105: 1, 5′ relates to interview number 105, page number 1, line number 5. This notation style is used in describing the results in relation to participants’ quotes.

All participants were provided with a summary of the results and invited to give feedback. All declined to review full transcripts of their interview.

### 2.4. Ethics Statement

The study was approved by the University of New South Wales Human Research Ethics Committee (UNSW HREC 08003) and the Aboriginal Health and Medical Research Council (AHMRC 615/07 and 1171/16). Community approval was provided by local elders who guided the project from its inception as well as by relevant local Aboriginal community-controlled organizations. Written informed consent was obtained from all participants included in the study.

### 2.5. Data Availability

The data that support the findings of this study contain potentially identifying or sensitive information that could compromise the privacy of the respondents and are, therefore, not publicly available. Data may, however, be available from the authors upon reasonable request with approval from participating communities and the AHMRC Ethics Committee.

## 3. Results

The study recruited a total of 34 participants: 20 from regional and 14 from urban communities. Participants included 10 males and 24 females, and all participants identified as being an Aboriginal person. Seven participants were aged 50–59 years old, 14 participants were 60–69 years old, and 13 participants were over 70 years of age.

### 3.1. Perceived Health Concerns

When prompted for the main health concerns of older people in their community, participants discussed a range of concerns, mainly under the themes of physical health, social and emotional well-being, and poor access to services (see Supplementary Material Table S2).

#### 3.1.1. Physical Health

##### Chronic Diseases

Susceptibility to chronic diseases and other health conditions are a persistent worry or fear for some with participants describing the worry as having a ripple effect across the community. A range of chronic diseases were identified, namely diabetes, cancer, cardiovascular disease, emphysema, and liver disease. Participants often raised concerns about experiencing multiple chronic diseases at once. Concern over the prevalence of smoking, alcohol abuse, poor diet, and obesity were also raised by many participants. Participants noted the prevalence of a “bad lifestyle” in the community (Participant 106: 1, 12) and urged that “they’ve got to stop drinking and smoking” (Participant 109: 1, 11). Poor health literacy appeared to play a role in health and ‘lifestyle’ choices for some participants.

##### Dementia

Among other chronic diseases mentioned by participants, dementia arose as a particularly significant concern. All participants spoke of the prevalence of dementia in the community with some identifying it as a new and growing concern.

“‘Onset of dementia’ is a term I’ve been hearing lately… and it’s not good, and they’re young, like, in their 50s” (Participant 113: 7, 3).

“Years ago, that (dementia) was never mentioned. (It was) cancer when I was growing up. And we’re living with it now. Dementia, we didn’t even know anything about it. And I suppose it’s been around for years” (Participant 107: 3, 14).

Participants also noted the significant impact that chronic disease and ageing have on caregivers with particular attention given to the strain that dementia places on caregivers. Participants commented on the perceived practical as well as emotional impact of taking on the responsibilities of a caregiver.

“Oh, I think it’d be very stressful. I don’t know how they (dementia caregivers) do it. Because it’s a 24–7 watch, isn’t it?” (Participant 101: 13, 17).

“It changes your lifestyle… They (the family) keep them home as long as they can… it’s more expense, more medication, going out to more appointments. I’ve just had a school friend, my age, and he was heartbroken; he just put his wife in a nursing home; terrible. The family have to make all these decisions, and financially, and it is, it’s very, very–it’s cruel and sad” (Participant 113: 7, 19).

##### Falls and Mobility

Participants also identified falls, pain, and loss of mobility as significant health concerns, with several reporting that they had fallen before. Falls and loss of mobility negatively affected the desired level of functioning for participants. As one participant explained:

“When the legs, the knees go on them or the hips, it’s really annoying …because your legs are so important to you…if you can’t use your legs, you’re an invalid. You’ve got to sit down all the time and that’s frustrating, that’s stressing. … Because we do everything when we walk. We go shopping, we stand in our kitchen, we stand in the shower” (Participant 105: 1, 5).

Others described the importance of mobility in maintaining their independence, quality of life, and participation in various physical, social, and cultural activities that they enjoy, such as dancing and long walks. Falls were described as having an impact on people “because it brings home the reality that they can’t keep doing things for themselves” (Participant 301: 3, 9).

#### 3.1.2. Social and Emotional Well-Being

The interviews included specific prompts regarding depression. However, participants’ responses generally focused on isolation and loneliness, grief and loss, and keeping health problems to yourself.

##### Isolation and Loneliness

Social isolation and loneliness were commonly described by participants, attributed to several factors. Many mentioned chronic health issues, poor mobility, and lack of transport as interconnected hindrances to the ability to leave the house and to care for themselves. Participants shared that the “restriction to do a lot of things they want to do” (Participant 104: 1, 6) results in isolation and a loss of independence. In another participant’s words, “Because we ain’t got a car. We can’t go visit anyone. If anyone’s sick, we’d like to go and see them” (Participant 102: 1, 3). Yet, even when out in the community, the rise of technological devices appears to have impacted older Aboriginal people by exacerbating the sense of isolation that they often feel.

“No-one’s talking to anyone anymore, you know … And they’re all there on this (mobile phone) ... (it impacts) especially older people. Because we used to all just talk to one another. But not today” (Participant 102: 43, 7).

Another participant shared that loneliness can still arise if opportunities for social contact are not suited to an individual’s needs and preferences, saying “Even when I went out with people, I was still lonely in a crowd. That’s probably why I used to and all that just because they drank and it was somewhere for me to go” (Participant 102: 25, 1). Notably, the lack of available or ongoing culturally safe group programs was also mentioned as a factor contributing to social isolation for older Aboriginal people.

##### Grief and Loss

Participants spoke openly of the difficulties related to bereavement and losing family members. They expressed the grief and loss they have experienced, and the effects on their mental health and well-being. As one participant described, “It takes a lot of coping with and getting over. It’s the hardest thing to lose someone in your family. It’s very hard” (Participant 013: 3, 19).

Participants also expressed how effects of the Stolen Generations and loss of land are with people today. Many participants spoke about their worry regarding the mental health of younger generations, including depression, anxiety, suicide, and drug use. Several participants identified the need for greater support and counselling to help people in the community cope with grief and loss. For example:

“Grief and loss that goes right through our society; actually, all over the years, all that’s happened to us. You know, the things we lose, and we don’t get proper counselling for it. A lot of us don’t get proper counselling once we lose a loved one” (Participant 008: 1, 2).

##### Health Issues Go Underground

Almost all participants indicated that community members tend to be private about their health problems. As one participant stated, “I don’t like asking anyone for help. I don’t like anyone knowing my business” (Participant 113: 2, 7). Sometimes the desire for privacy was related to feelings of shame or stigma. Participants also commonly alluded to a stoic attitude that they adopt to downplay or normalize their own difficulties.

Participants explained that older people feel worried about being a burden or dependent on their family, especially their children who have “probably got their own lives to live” (Participant 110: 6, 15). Participants observed that this is why they tend to “wait ‘til they collapse before they go (to a health service)” (Participant 102: 10, 12) or had “kept that to myself” (Participant 115: 2, 2) in the past.

Depression, in particular, is a health issue that is often not heard about in the community because people “cover it up” (Participant 115: 2, 13) or “keep their masks up” (Participant 301: 3, 1). One participant spoke of seeking counselling treatment outside their community, to maintain their privacy (Participant 113: 9, 1). Others explained that community members do not always disclose their concerns to health professionals. In the words of one participant, “I think you can’t see it, though people don’t want to do this or that anymore, but tell the doctors ‘no, I don’t have depression’” (Participant 015: 3, 20).

#### 3.1.3. Access to Healthcare Services

A few participants raised the issue of poor access to health services when prompted for the main health concerns of older people in the community. This was discussed in terms of the shortage of medical staff, distance to services and transport difficulties, and insufficient outreach and awareness of available services.

Participants also reported on the difficulty older people face when accessing services through technological platforms. As one participant reported, “a lot of older people don’t know how to access” services (Participant 104: 19, 10), and explained that being competent with the use of technological devices enabled some to “know all about the services” and to “use everything that’s there” but that many people are not aware of what is available, especially because “they don’t like using their mobile phones” (Participant 104: 32, 6). Telephone access could also be a problem, with one participant reporting that they were left “hanging on the phone for ages ... So, I just give them my number and that to ring me and well, I haven’t heard from anyone” (Participant 102: 44, 18).

Several participants also explained that there are differences in communication styles between Aboriginal and mainstream health service providers, highlighting the importance of cultural safety in healthcare. As one participant put it, Aboriginal people are often “too afraid to look into a big, well-educated person’s face” (Participant 105: 20, 8). There was acknowledgement of the comfort provided by Aboriginal staff at the local hospital: “you’re not alone, you’re not by yourself, someone’s there looking out” (Participant 016: 1, 2). In a similar vein, one participant also voiced concern over social inequity and discrimination when talking about barriers to participation in health programs, saying that “we’ve been blocked off ... there’s people who can pay for those cures where poor, old, black people, we cannot, so therefore we die, they live” (Participant 105: 22, 17).

### 3.2. Participation in Current Health Programs

Participants discussed the barriers and enablers of participation in current community health programs (see Table 1). An apparent lack of suitable programs, transport constraints, shame and stigma, hopelessness towards the challenges of ageing, as well as disengagement due to past negative experiences, including “political reasons” (Participant 114: 12, 9) have emerged as barriers. At the same time, enablers included the flexibility of attendance, social aspects, and availability of transport.

### 3.3. Strategies to Activate Readiness to Adopt Healthy Ageing Programs

Participants were prompted to describe what a culturally-appropriate, healthy ageing program should look like. Responses centered on cultural safety and the incorporation of various activities into a holistic program. Themes emerging from the interviews further suggested that family, community, cultural identity, and empowerment regarding ageing were important considerations in designing a healthy ageing program.

#### 3.3.1. Culturally Safe Care

##### Aboriginal-Specific Program

Many participants described how they did not attend any mainstream local community health programs, preferring to attend local Elders’ groups where they are together with other Aboriginal people they have known for a long time.

##### Aboriginal or Culturally Responsive Staff

Participants expressed a desire for “the right people in the right services that cater for us in the way that we should be catered for” (Participant 104: 6, 6). As another participant similarly explained,

“Make sure that the instructor or whoever the organizers are, that they have the same ideas as us, and that… they have an understanding of Aboriginal culture ... they’ve got to know where we’re coming from as well as know what they’re doing” (Participant 113: 12, 2).

##### Accessible and Culturally Secure Location

“The main thing is the venue. You’ve got to have it where everyone is, and then invite everyone and make them feel welcome” (Participant 113: 4, 10).

#### 3.3.2. A Holistic Program

**Figure 1:**
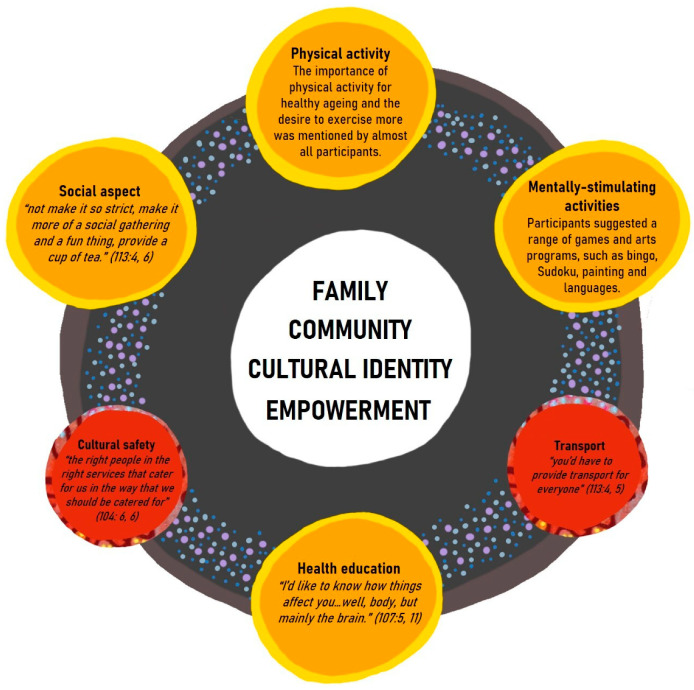
Model for a holistic, healthy ageing program.

Overall, participants suggested four main activities of which a healthy ageing program should comprise. This includes physical activity, mentally-stimulating activities, social activities, and health education, as described in Figure 1. Participants spoke about a range of different activities that they and other older Aboriginal people in their community enjoy, such as walking, fishing, painting, and socializing with friends. The central tenets of family, community, cultural identity, and empowerment, which are discussed in the sections below, are visually illustrated to be at the heart of the program. The necessity for cultural safety and transport is represented with the red circles in Figure 1. Participants also suggested that involving younger people was important, along with designing a program in which people “feel they’re not being judged” (Participant 301: 1, 19).

##### Family and Community

Numerous participants described the value of a supportive culture within their communities. One participant explained that, “When anything happened… it’s all the families that come together… They drop everything just to come down and support those families in grieving. That’s always been the culture up home where I come from” (Participant 105: 14, 11). Participants also expressed the responsibility they feel over the younger generations–the need to mentor them as well as the desire to pass on cultural knowledge and values; that is: “…getting them under my wing and try and talk to them” (Participant 107: 11, 15).

Several participants volunteered their views on the topic of respect and reciprocal care, further emphasizing the importance of intergenerational connections. Participants observed a loss of respect for older people from younger generations and expressed a desire for their families, especially the younger ones, to help them more. Some indicated that they were frustrated and angry about contemporary attitudes towards older people, which can sometimes result in elder abuse.

“If we can get the younger people back on board to show–to give more concern for the older people, things might work out better… it’s a matter of the younger people having a bit more respect for their older people” (Participant 104: 6, 12).

“Some of the Elders don’t cope well because they haven’t got the support that they need from their family… I think families, from what I observe, get frustrated with their elders and sometimes it ends up being verbally abused, physically abused, or lack of respect for the older person” (Participant 301: 2, 6).

##### Empowerment Regarding Ageing Well

Many participants expressed a positive outlook on ageing, strong motivation, and belief in their capacities to improve their own health and well-being. As one participant explained: “We’re not young anymore, we’re old and we have to learn how to cope with being old and there’s lots of things that you can do” (Participant 105: 2, 7).

There was a sense of pride and self-respect expressed by a number of participants regarding the way they had already made positive lifestyle changes (e.g., engaging in physical exercise or quitting smoking) and seen the resulting benefits in terms of their health and well-being.

“…I said, ‘I’m done with drinking, I’m done with smoking.’ Oh, I feel a lot better, I would never do it–never look at (smoking or drinking) now” (Participant 109: 3, 4).

“At the beginning I hated it, but then I felt good. I was on the walker, I was doing everything with the balls, and I was going up” (Participant 113: 3, 14).

### 3.4. Technology

Finally, we asked participants about their attitudes towards the use of technology, to explore the acceptability of using technology as a platform for the delivery of a healthy ageing program with older Aboriginal people. A majority of participants were familiar with and use technological devices in their everyday lives to contact others or to play games. Many were receptive to using technology, felt technically competent, and could see the benefits of using technology in terms of being “in touch with the rest of the world” (Participant 104: 26, 10) and keeping their minds active. As described by one participant, “I get on the computer and do all my own loans, funds, change of address, whatever I want to do… your brain’s clicking over all the time” (Participant 113: 12, 14).

On the other hand, some participants described a reluctance to engage with technology, saying for example, “I can’t imagine myself sitting there playing with (a computer) … That would, I think, would just annoy me. It’s just not me” (Participant 106: 6, 5). Alternatively, some felt that older people would struggle in terms of technical capability. “For older people it’s very confusing. They don’t know how to use them things … they can’t seem to grasp it” (Participant 104: 20, 15). However, several participants who were not confident in their technical competence, expressed an interest in learning how to use devices, especially if it was made simple and they were given adequate support.

## 4. Discussion

The current study found that older Aboriginal Australians are concerned about chronic health conditions, loss of mobility, social and emotional well-being, and difficulties accessing services. A range of barriers and enablers to participating in current health programs was also identified. From the perspective of older Aboriginal people, a successful model of a healthy ageing program is one that is culturally safe, holistic, and responds to their health concerns and previous experiences. For many, the use of technology was also a viable approach to the delivery of health programs.

### 4.1. A Co-Designed Healthy Ageing Program

Echoing the available evidence [14,15,16], participants were keen to engage in healthy ageing programs, but current programs do not meet their needs. Findings on the apparent lack of suitable programs and disengagement due to previous negative experiences demonstrate the failure of programs to meet the community’s needs and emphasize that a co-design approach is essential. There is a need for direct input from the community regarding program content to ensure it is relevant to participants and to foster a sense of ownership over the program [16,20].

Based on this study’s findings, a health program that addresses the relevant health priorities of community members, overcomes barriers to participation in existing programs, and incorporates various enablers for participation can be developed.

### 4.2. Cultural Safety

The necessity for culturally safe care, such as having Aboriginal-specific programs, Aboriginal or culturally proficient staff, and a culturally safe venue in the community, was frequently raised. To effectively engage with older Aboriginal people and achieve health promotion goals, culturally responsive approaches are imperative [43,44,45,46].

Findings show that shame and stigma result in many older Aboriginal people concealing their health problems and deter some from participating in health programs. Various studies have reported on shame and stigma as barriers to positive health seeking behaviors among Aboriginal people in the context of mental illness, hepatitis C, or cancer [47,48,49]. This highlights the need for a strengths-based approach, which focuses on positive health and well-being for all older Aboriginal people rather than on deficits, and is culturally appropriate, socially inclusive, and empowers individuals to engage in positive health behaviors.

### 4.3. Family, Community, and Cultural Identity

The NAHS definition of Indigenous Health includes the ‘well-being of the whole community’. This was reflected by participants frequently referring to their community when discussing their personal health. Community and strong kinship ties are core features of the cultural identity and collective perspective that Aboriginal people have [50]. This represents a significant divergence from the mainstream individualistic approach to healthcare [51].

Findings also highlighted that performing the role of mentor to younger generations was significant for many older Aboriginal people, as identified in previous studies [52,53]. However, at the same time, participants expressed concern that the degree of respect and assistance that older people receive from younger generations has deteriorated. Aboriginal families and communities are now seeing a lack of the traditional reciprocity of care between individuals [54]. A co-designed healthy ageing program should aim to build self-identity and empower older Aboriginal people to continue fulfilling these cultural roles. Community-building activities and opportunities for intergenerational connections, such as those that involve younger people in cultural activities can be incorporated [50].

### 4.4. A Holistic Healthy Ageing Program

Consistent with literature on the prevalence of multi-morbidity among Aboriginal Australians [3], the community-level concern over multi-morbidity was demonstrated in this study. Whilst all participants discussed health in relation to their physical status, most participants extended their discussion on health and ageing beyond physical health to include their social and emotional well-being, cognitive health, and even their access to health services. This multi-faceted concept of health is consistent with the NAHS definition of Indigenous Health and needs to be incorporated into health programs to meet the needs and expectations of older Aboriginal people. In this regard, a holistic health program, which targets various aspects of health such as physical, mental, social, and health literacy, as suggested by participants, would be appropriate. Notably, there are various existing programs and services in these Aboriginal communities, which can be linked with such a holistic healthy ageing program to increase uptake and improve health outcomes and behavioral change [55].

### 4.5. Cognitive Training

Specific concerns regarding dementia risk were apparent in this study, along with a desire for healthy ageing programs to incorporate activities focused on optimizing and maintaining cognitive functioning. Dementia is three to five times more prevalent among Aboriginal than non-Aboriginal Australians [56]. Furthermore, Aboriginal people experience many risk factors for dementia, including cardiovascular disease, diabetes, and tobacco use at higher rates than non-Aboriginal people [3]. There is evidence that suggests cognitive training interventions can improve cognitive performance in healthy older adults [57,58]. There are a variety of cognitively stimulating activities, particularly those that also entail aspects of social or physical activity, which are also likely to provide benefits for brain and cognitive health in later life [59,60,61,62].

### 4.6. Cultural and Social Connectivity

Notably, the arts such as painting and Aboriginal language programs arose as suggestions to be incorporated into a healthy ageing program. Ware [55] reports that participation in arts programs can benefit Aboriginal people by improving physical and mental health and well-being as well as fostering greater social cohesion. The incorporation of such arts programs would support the cultural identity of older Aboriginal people and participants’ desires for cognitively stimulating activities and social interactions in a healthy ageing program.

### 4.7. Health Education and Empowerment

Participants expressed a desire for health education to be a component of a healthy ageing program. Research has shown that Aboriginal understanding of diseases can deviate significantly from western biomedical explanations [63,64] and that low health literacy negatively affects health outcomes and patient safety [65]. Supporting Aboriginal people in understanding the biological basis of diseases by increasing access to health information can effectively enhance health awareness and health-seeking behaviors and empower Aboriginal people in making informed health and lifestyle decisions [63,66]. Such health empowerment would be crucial in response to participants’ feelings of hopelessness and resignation toward the poor prospects of old age.

### 4.8. Using Technology to Deliver a Healthy Ageing Program

Some participants described experiencing an increased sense of isolation as a result of the widespread use of technology in modern society. Yet, many participants were interested and willing to use technological devices. Several participants discussed their concerns over the limited technical proficiency older people face, but also expressed a willingness to learn to use technological devices. There is a growing body of literature indicating various technologies provide opportunities to mitigate loneliness and social isolation through social inclusion, engagement in activities of interest, and boosting self-confidence [67,68]. It is suggested that health care providers should introduce older people to appropriate technologies to help overcome the issue of social isolation [68].

Notably, as highlighted in our model for a holistic healthy ageing program, the use of technological platforms should not be a total substitute for in-person contact. However, this study contributes to the evidence for the acceptability of technology-based health programs among older Aboriginal people [27,28]. This is in contrast to the common impression that older people tend to be less technologically-competent and less accepting of its use [69]. The barrier of having lesser technical ability can be overcome by designing app characteristics, such as graphics and ease of use, to accommodate older people [29,70,71]. When designed to support Aboriginal people’s needs, technology-based health interventions could add an important element to improving the healthy ageing of Aboriginal people.

### 4.9. Study Limitations and Suggestions for Future Research

The authors acknowledge that this project is relevant to the participating communities and the findings may not generalize to other Aboriginal communities. There was also a notable three-year gap between the regional and urban interviews, which could have had a small impact on the findings. Other limitations included a relatively small volunteer sample with recruitment from only one regional and one urban area. There was also the potential for interviewer characteristics, such as non-Aboriginality or Aboriginality and familiarity, to have influenced participants’ responses.

Following this study, we aim to apply these research findings in the co-design of a culturally-appropriate, healthy ageing program, which is exercise-based and capitalizes on technology as a delivery platform. The implementation and evaluation of such a co-designed healthy ageing program among older Aboriginal Australians should be explored in future studies.

## 5. Conclusions

This study has established that the needs of older Aboriginal people as they age are complex, emphasizes the value of the collaborative development of health programs for Aboriginal people, and has identified meaningful aspects to be incorporated into a culturally-responsive, healthy ageing program. These aspects include cultural safety, a holistic program which comprises physical activity, cognitive training, social interaction, and health education, and which maintains family, community, cultural identity, and empowerment regarding ageing well as its central tenets. This study also provides preliminary evidence for the use of technology as a viable platform for the delivery of health programs.

## Figures and Tables

**Table 1:** Barriers and enablers of participation in current community health programs.

Barriers	Enablers
Lack of programs “We got nothing here for older people” (Participant 104: 5, 14).	Flexibility of attendance “I have a choice. I can say one day, “I don’t feel like going” (Participant 113: 11, 1)
Unappealing programs Participants describe programs to be “boring” (Participant 106: 15, 6) (Participant 101: 21, 17), or are “too long” (Participant 111: 4, 11), or run by ineffectual staff who “need to lift their game” (Participant 106: 20, 18).	Social aspect “The camaraderie, they’re going to make you feel better in yourself. Health-wise, they can only help as well, but it’s just meeting and talking to everyone” (Participant 113: 10, 19). “It’s good fun…And you have a laugh and all that with them. And yeah, mainly just to get out the house for a couple of hours” (Participant 102: 40, 7).
Transport constraints Several participants noted that having to “find your own way” (Participant 111: 4, 16) or “getting someone to pick you up” (Participant 113: 12, 2) pose barriers.	Transport readily available They “always (have) transport” to programs (Participant 102: 41, 8) (Participant 112: 4, 19). “Transport around here is terrific” (Participant 101: 22, 8)
Shame or stigma associated with participation “Shame would be a big factor (barrier). Shame to admit that we ourselves are facing those crises within our lives. Shame of what the community may think about us.” (Participant 003: 5, 14)	
Disengagement associated with past experiences with programs or other program attendees. “I’ve seen it all, we’ve already been there and done that, tried to help, you know?” (Participant 110: 3, 1) “I want to find something better now than last time not with them–all them whinging and moaning all the time and they got everything.” (Participant 102: 38, 12)	
Sense of hopelessness or disempowerment regarding ageing well. Participants expressed feelings of hopelessness towards the challenges of ageing and were resigned to the inevitability of disease and poor prospects of old age. “We didn’t think it (growing old) was going to be so hard for us.” (Participant 104: 7, 4) “Well, you just live with it (chronic disease). What else can you do? You can’t do anything.” (Participant 110: 4, 18) Some suggested that health outcomes are a gamble, where taking better care of one’s health “doesn’t always pay off” (Participant 301: 3, 9).	

## Supplementary Materials

The following are available online at https://www.mdpi.com/1660-4601/17/20/7390/s1, Supplementary Material Table S1: Semi-structured interview questions. Supplementary Material Table S2: Coding and themes.
