# Supplementary material for: Older Aboriginal Australians’ Health Concerns and Preferences for Healthy Ageing Programs
Source: Int J Environ Res Public Health. 2020 Oct 10;17(20):7390. doi: 10.3390/ijerph17207390 (PMC7600369; doi:10.3390/ijerph17207390)
Supplement: Supplementary file 1 [file ijerph-17-07390-s001.pdf]

**Supplementary Material Table S1: Semi-structured interview questions**

1. What do you see as the main health concerns for older people in your community?

    1.1 What do you think your chances are of experiencing any of these?

2. What do you think you do well towards your health?

    2.1 What do you think you could do better towards your health?

3. If there was a healthy (brain?) ageing program which was culturally appropriate and relevant to your community, what would it look like?

4. How often do you hear about someone having diabetes, high blood pressure, heart disease or obesity?

    4.1 How do these health issues impact on a person and their family's lives?

    4.2 What about dementia or memory problems, how often do you hear about someone having dementia or memory problems?

    4.3 How does dementia or memory problems impact on families and individuals lives?

    4.4 What about depression, how often do you hear about someone having depression?

    4.5 How does depression impact on families and individuals lives?

    4.6 How does the concern or worry about falls impact on families and individuals lives?

*One of the planned outcomes from this project is to develop a range of health intervention programs. These may include healthy eating, lifestyle and home exercise programs and also brain training using ipad and personal tablet computers.*

5. Would you be interested in participating in a community workshop/focus group where you will be shown some brain and health training programs and asked to share your thoughts about them?

6. If any of the programs were developed, would you be interested in participating?

7. Do you currently participate in any community health programs?

    7.1 Which programs are they?

    7.2 What is it about these programs that you like/dislike?

    7.3 What do you see as the barriers to participating in community health programs?

8. Do you use a smart phone, or another device like an iphone, ipad, tablet or computer?

    8.1 Which devices are you familiar with?

9. Is there anything else you would like to add?

**Supplementary Material Table S2: Coding and themes**

**Table 1.** Themes that emerged from regional interviews.

| Core category | Themes | Sub-themes |
|---|---|---|
| 1. As well as chronic diseases, and fears associated with falls, depression, dementia and anxiety are a constant worry | 1.1 Shame associated with depression<br>1.2 Mental health issues go underground | 1.1.1 Linked to grief and loss<br>1.2.1 Need to address the effects of suicide<br>1.2.2 Alcohol and other drugs |
| 2. Necessity for Culturally Safe Care | 2.1 Comfortable with Aboriginal Support Staff<br>2.2 Positive Regard for Aboriginal Medical Service (AMS) | 2.1.1 Cultural Considerations for Healthy Ageing Programs<br>2.1.2 Preference for Elders groups |
| 3. Considerable motivation for improving health and wellbeing | 3.1 Much support for co-design research project and support for program development<br>3.2 Health related knowledge gaps | 3.1.1 Missed opportunities and knowledge gaps - for example technology |

**Table 2.** Themes that emerged from urban interviews.

| Core category | Themes | Sub-themes |
|---|---|---|
| 1. Healthy ageing priorities | 1.1 Chronic conditions and physical functioning<br>1.2 Social and emotional wellbeing<br>1.3 Access to services | 1.1.1 Dementia is a significant concern<br>1.2.1 Loss of independence<br>1.2.2 Isolation and loneliness<br>1.2.3 Grief and loss<br>1.2.4 Keeping health problems to selves<br>1.1.2 Inequity |
| 2 Attitudes towards Ageing | 2.1 Hopelessness and despair<br>2.2 Motivation | |
| 3 Family and community | 3.1 Carer wellbeing<br>3.2 Community support<br>3.3 Intergenerational relationships | |
| 4 Barriers and enablers of a healthy ageing program | 4.1 Lack of programs<br>4.2 Wearied<br>4.3 Transport<br>4.4 Aboriginal or culturally responsive staff<br>4.5 Holistic program | 4.5.1 Physical activity<br>4.5.2 Mentally stimulating activities<br>4.5.3 Social activities<br>4.5.4 Health education |
| 5 The use of technology | 5.1 Receptivity<br>5.2 Resistance | |

**Table 3.** Overall themes from amalgamated data.

| Core category | Themes | Sub-themes |
|---|---|---|

| | | |
|---|---|---|
| 1. Perceived health concerns and healthy ageing priorities | 1.1 Physical health<br>1.2 Social and emotional wellbeing<br>1.3 Access to healthcare services | 1.1.1 Chronic disease<br>1.1.2 Falls and mobility<br>1.1.3 Dementia<br>1.3.1 Isolation and loneliness<br>1.3.2 Grief and loss<br>1.3.3 Keeping health problems to selves<br>1.3.4 Concern for youth |
| 2. Barriers and enablers to participation in current healthy ageing programs | 2.1 Lack of programs<br>2.2 Transport<br>2.3 Culturally safe care<br>2.4 Failure to meet needs and preferences | 2.3.1 Aboriginal-specific program<br>2.3.2 Aboriginal or culturally responsive staff<br>2.3.3 Culturally safe location |
| 3. Strategies to activate readiness to adopt healthy ageing programs community | 3.1 Culturally safe care<br>3.2 Holistic program<br>3.3 Family, community and cultural identity<br>3.4 Empowerment regarding ageing well | 3.2.1 Physical activity<br>3.2.2 Mentally stimulating activity<br>3.2.3 Social activities<br>3.2.4 Health education |
| 4. The use of technology | 4.1 Receptivity<br>4.2 Resistance | |
